# Piezotronic Effect: An Emerging Mechanism for Sensing Applications

**DOI:** 10.3390/s150922914

**Published:** 2015-09-11

**Authors:** Kory Jenkins, Vu Nguyen, Ren Zhu, Rusen Yang

**Affiliations:** Department of Mechanical Engineering, University of Minnesota—Twin Cities, 111 Church Street SE, Minneapolis, MN 55446, USA; E-Mails: jenk0131@umn.edu (K.J.); nguy1993@umn.edu (V.N.); zhuxx453@umn.edu (R.Z.)

**Keywords:** piezotronic, strain sensor, pressure sensor, gas sensor, biosensor, chemical sensor, photodetector, temperature sensor, zinc oxide, nanowire

## Abstract

Strain-induced polarization charges in a piezoelectric semiconductor effectively modulate the band structure near the interface and charge carrier transport. Fundamental investigation of the piezotronic effect has attracted broad interest, and various sensing applications have been demonstrated. This brief review discusses the fundamentals of the piezotronic effect, followed by a review highlighting important applications for strain sensors, pressure sensors, chemical sensors, photodetectors, humidity sensors and temperature sensors. Finally, the review offers some perspectives and outlook for this new field of multi-functional sensing enabled by the piezotronic effect.

## 1. Introduction

Novel technologies for sensing electronics with multi-functionality and enhanced performance are important for emerging applications such as wearable electronics, robotics, prosthetics, and biomedical treatments. Piezoelectric materials have long been used for actuator, energy, and sensing applications. The piezoelectric nanogenerator fabricated from zinc oxide (ZnO) nanowires [1,2,3,4,5,6,7,8] inspired wide interest in piezoelectric nanomaterials and enabled new applications such as self-powered nanosensors [9,10,11,12]. ZnO nanowires are also semiconducting and suitable for developing electronics. The coupling between the piezoelectric and semiconducting properties of nanowires was discovered in 2006 [13], in which the strain-induced piezoelectric polarization was used to tune the conductivity of the nanowire. This effect was called the piezotronic effect, and has been investigated in piezoelectric, semiconducting nanomaterials like ZnO, zinc sulfide (ZnS), cadmium sulfide (CdS), indium nitride (InN), gallium nitride (GaN), and monolayer molybdenum disulfide (MoS_2_) [14,15,16,17,18,19,20]. Mechanical stimuli are ubiquitous and abundant in the environment. The piezotronic effect combines piezoelectric polarization with semiconductor properties and allows the direct and active interaction between devices and stimuli. This new fundamental phenomenon continues to inspire novel device applications and has led to an emerging field called “piezotronics”.

The piezotronic effect was originally applied for strain sensing [13,21], and significant progress has been made in developing other sensors and novel electronic devices with much enhanced performance. For instance, the adsorption of chemical species onto a semiconductor material acts as a gate voltage, tuning the channel conductivity across the bulk material. This phenomena has been widely used for chemical sensing [22,23]. When the sensor includes piezoelectric semiconductor materials and a Schottky junction at the metal–semiconductor interface, the voltage from the adsorbed species can alter the energy barrier height and regulate the carrier transport. At the same time, the barrier can also be tuned with the strain-induced piezoelectric potential [20]. The piezotronic effect can thus be used for enhancing the performance of chemical sensors [24].

Nanostructure-based piezotronic sensors have attracted much attention because of their low power consumption and high sensitivity enabled by large surface area to volume ratio and novel piezotronic effect. Although there are many excellent reviews discussing piezotronics [17,25,26], to the best of our knowledge, assessments of the various sensing applications are still limited [27]. This article aims at reviewing exclusively the fundamentals of piezotronics and recent applications for typical sensors, including strain sensors, pressure sensors, chemical sensors, photodetectors, humidity sensors, and temperature sensors.

## 2. Fundamentals of Piezotronic Effect

### 2.1. Piezoelectric Effect

Piezoelectricity exists in non-centrosymmetric crystalline materials. In a piezoelectric lattice, mechanical stress alters the distance between the center of positive charges and the center of negative charges, which creates electric dipole moments or changes the existing ones. In either way, polarization charges are induced on the surface of the material. Alternatively, an electric field can cause mechanical strain in the crystal. Such an electromechanical interaction has found applications in the industries of sensors, actuators, and transducers [28].

Recently, many piezoelectric nanostructures have been synthesized, including ZnO [29], CdS [30], ZnS [31], GaN [32], InN [33], poly(vinylidene fluoride) (PVDF) [34], diphenylalanine peptide [35], lead zirconate titanate (PZT) [36], barium titanate (BaTiO_3_) [37], sodium niobate (NaNbO_3_) [38], lithium niobate (LiNbO_3_) [39], tellurium (Te) [40], few-layer MoS_2_ [41], *etc*. The unique properties of nanomaterials have advanced many research areas, and one of the most fruitful fields is sensing technology based on or enhanced by the piezotronic effect [27].

### 2.2. Piezotronic Effect

The piezotronic effect was first discovered in 2006 in piezoelectric ZnO nanowires with n-type conductivity in Wang’s group [13]. Generally, it exists in heterojunction systems with one material being a piezoelectric semiconductor and the other being a metal, a semiconductor, or an electrolyte [42]. When the piezoelectric material is deformed, polarization charges are induced at the junction between two materials, which modify the interfacial band structure and thus the carrier transport, trapping, generation, and recombination processes. In short, piezotronic effect is a change of the interfacial carrier dynamics due to the piezoelectric polarization.

**Figure 1:**
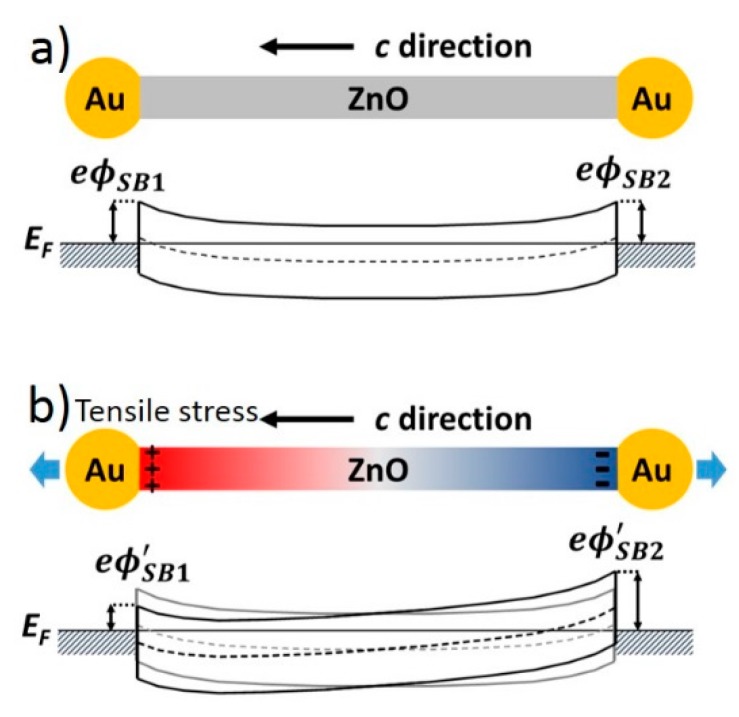
Schematic band diagram of a ZnO nanowire forming Schottky contact with Au electrodes. (**a**) Band diagram in the absence of strain, $e{\text{ϕ}}_{SB1}$ and $e{\text{ϕ}}_{SB2}$ are Schottky barrier height at the two contacts; (**b**) Band diagram in the presence of tensile strain. Positive piezoelectric charges at the left contact reduce the barrier height to $e{\text{ϕ}}_{SB1}^{\prime }$, and negative piezoelectric charges at the right contact increase the barrier height to $e{\text{ϕ}}_{SB2}^{\prime }$. The gray lines represent the original band diagram without strain.

The most studied piezotronic material system consists of a ZnO nano/microwire and metallic electrodes, and is used as an example here to explain the piezotronic effect. As shown in Figure 1a, a ZnO wire has its crystallographic c-direction pointing left and two gold electrodes on its two ends. As-synthesized ZnO wires usually have n-type conductivity with intrinsic defects and impurities as shallow donors [43], and it forms Schottky barriers with high work function metals, such as gold. Schottky barrier is a rectifying contact. It allows only electron transport from ZnO to gold and prevents the electron transport from gold to ZnO. Two back-to-back Schottky diodes result in very little current flow. In Figure 1b, the ZnO wire is under tensile stress, and positive polarization charges appear on the left end and negative charges on the right. Polarization charges will be partially compensated by internal and external free carriers, but may not completely diminish due to the moderate doping level and finite charge screening lengths of electrodes [44]. At steady state, remnant piezoelectric charges still exist at the two contacts, and the electrostatic field from those positive charges reduces the Schottky barrier height, while negative charges raise the Schottky barrier height. The asymmetric barrier change makes it easier for electrons to transport from left to right. Similarly, a compressive stress induces piezoelectric charges with opposite polarities, and allows electron flow from right to left.

Several features of the piezotronic effect are emphasized here. First, fundamental theory shows that the change of the Schottky barrier height is proportional to the piezoelectric charge density, which is proportional to the strain. Because the current depends exponentially on the Schottky barrier height, the relationship between strain and current flow is also exponential [20]. Second, the piezotronic effect is not a transient effect. As long as the strain holds, remnant piezoelectric charges can stay at the interface and the piezotronic effect will not disappear [44], although some slight decay over time was also observed [21]. Third, the piezotronic effect is an interface phenomenon, and should not be confused with the piezoresistive effect. The piezoresistive effect describes a change of the electrical conductivity of a semiconductor or metal when strain alters its bandgap, and thus it is a volume effect. Usually, the piezotronic effect has a more significant influence on the current flow than the piezoresistive effect when a Schottky barrier exists [45].

### 2.3. Piezotronic Material

A material must be both piezoelectric and semiconducting in order to demonstrate the piezotronic effect. Despite the strict requirements, the family of piezotronic materials is not a small one, and it includes some of the most important electronic materials. The piezotronic effect was first observed in wurtzite ZnO and GaN [46], both having optoelectronic applications. Other wurtzite crystals like CdS and InN have similar properties and show the piezotronic effect as well. Few-layer MoS_2_ is an emerging two-dimensional semiconductor, and recently the piezotronic effect has been reported in monolayer MoS_2_ prepared by exfoliation or chemical synthesis [19,47]. Other transition metal dichalcogenide monolayers with semiconductivity and predicted piezoelectricity, such as MoSe_2_ and WS_2_, may also be candidates for fabricating piezotronic devices [48]. Conventional ferroelectric ceramics have very high piezoelectric coefficients, but they are insulators. However, with impurities acting as a dopant, BaTiO_3_ becomes a semiconductor [49]; exploration of its potential in piezotronics may possibly be an interesting research topic.

## 3. Piezotronics for Sensing Applications

### 3.1. Piezotronics for Strain Sensing

Applications for strain sensing are numerous including structural health monitoring, MEMs/NEMs devices, and human-computer interaction [50,51]. Piezotronic devices are especially suited for strain sensing due to the inherent reliance of the piezotronic effect on strain-generated piezopotentials to mediate charge transport across the electrode-semiconductor heterojunction [20]. Consequently, strain sensors make up a significant portion of current piezotronics sensing literature. We will take this opportunity for a departure from the traditional review format by first discussing some illustrative examples in detail, followed by a discussion of emerging themes in piezotronic strain sensing. The overarching themes of material system, device architecture, characterization, and performance will be explored while highlighting novel and unique approaches in various works.

**Figure 2:**
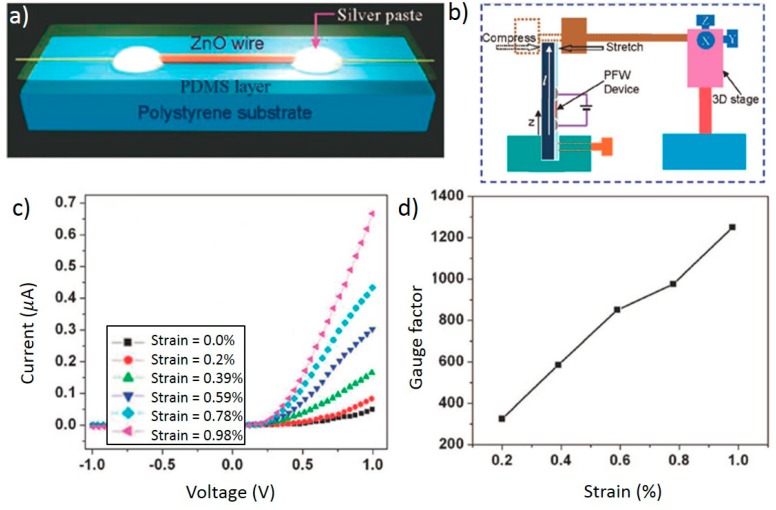
Concept illustration of the completed device (**a**) and strain testing setup (**b**); I-V characteristic curves for varying strains (**c**); (**d**) Gauge factor for various strains. Adapted with permission from [21]. Copyright 2008 American Chemical Society.

Strain sensors built on single ZnO nanowires are well represented by Zhou *et al.* [21]. Here, a single, lateral ZnO nanowire strain sensor on a polystyrene (PS) substrate is reviewed. Several devices were fabricated in this study, with nanowires grown by thermal evaporation. The nanowires ranged from 2 to 6 μm in diameter with lengths ranging from hundreds of microns to millimeters. The single wire was transferred to a flexible polystyrene substrate (3.0 cm × 0.5 cm × 0.1 cm) and connected to the substrate on each end using silver paste as shown in Figure 2a. The silver formed Schottky contacts at the source and drain electrodes and the entire device was encapsulated in PDMS for protection.

Testing was accomplished by fixing one end of the device in a holder and attaching the other end to a moveable stage as illustrated in Figure 2b [21]. Based on the geometry and mechanical properties of the device, the strain was assumed to be purely in tension or compression in the c-direction of the wire. Using the stage to apply strains ranging from 0% to 0.98%, the I-V characteristics were mapped over a bias of −1 V to +1 V and are presented in Figure 2c. As expected, the behavior was highly nonlinear due to the Schottky contact between ZnO and silver. The device is highly stable as demonstrated by periodic strain testing at 2 Hz and a fast response time of 10 ms was measured. Lastly, the gauge factor of the device is characterized in Figure 2d. The highest reported value was 1250, which the authors found to be higher than the gauge factor of 850 achieved for a piezoresistive carbon nanotube (CNT) strain sensor [21,52].

**Figure 3:**
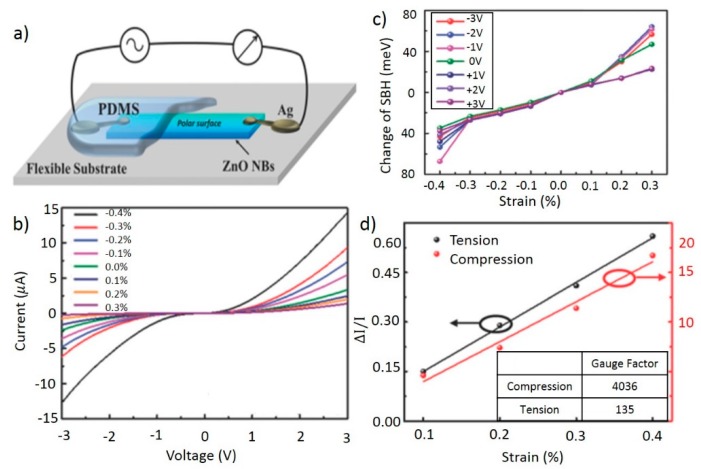
(**a**) Schematic diagram of the device; (**b**) Current-voltage curves as a function of strain; (**c**) Schottky barrier height change as a function of bias for varying strains; Inset shows gauge factor for tensile and compressive cases (**d**). Adapted from [53] with permission of The Royal Society of Chemistry. Copyright 2015.

Nanobelts are another common morphology for nanostructured strain sensors, as evidenced by Zhang *et al.* [53]. Unlike comparable nanowire devices, the source and drain electrodes for this sensor were connected to the same monopolar surface of a ZnO nanobelts as shown in Figure 3a. Doped with indium for improved mechanical performance, the nanobelt was fixed on a flexible PS substrate and packaged with PDMS. The nanobelt dimension were approximately 20 μm long by 180 nm wide. Identification of the polar direction is not required thanks to the monopolar surface configuration, simplifying device fabrication.

The device was placed under strain by fixing one end and applying bending [53]. A bias voltage was swept from −3 V to +3 V and the current output was then measured to map the I-V curves for strains from −0.4% to 0.3% as seen in Figure 3b. The change in Schottky barrier height for a given strain is shown in Figure 3c as a function of bias. Normalized current *vs.* strain and gauge factor under various strain conditions are presented in Figure 3d for both tension and compression. A gauge factor of 4036 was reported for compressive strain, which is the highest reported gauge factor in the strain sensors that we reviewed. For tensile strain, a gauge factor of 135 was reported. The difference in gauge factors for tensile and compressive strain was hypothesized to be caused by screening from increased electron mobility due to the piezoresistance effect. A response time of 120 ms was measured for the device under periodic compressive and tensile strains of varying intensities.

The previously reviewed strain sensing devices represent characteristic qualities of piezotronic strain sensors. In the following sections, we will summarize some common trends in piezotronic strain sensor design while highlighting novel and notable exceptions to design, materials, characterization, and performance.

#### 3.1.1. Materials and Morphology

Materials which are both piezoelectric and semiconducting are excellent candidates for piezotronic strain sensors. Nanowires are by far the most commonly used structures, with nanobelts and thin films also being explored [21,46,50,51,53,54,55,56,57,58]. Nanowire-based devices may be either single nanowire or arrays of many nanowires [21,50]. Single nanowire devices allow for very high sensitivity, while nanowire arrays provide robust and stable devices due to the redundancy of multiple wires [50,54]. In the case of Liao *et al.* [56], ZnO nanowires were combined with carbon fibers to form a hybrid strain sensing structure, demonstrating the potential of fabric-based piezotronic devices. Typically, nanowires are oriented laterally on a flexible substrate, with the bending applied perpendicular to the direction of the wire [21,51,58]. Uniquely, Zhang *et al.* makes use of arrays of vertical ZnO nanowires, perpendicular to the substrate, improving device resilience and achieving a gauge factor higher than previously reported for a single nanowire [21,50]. While lateral nanowires technically experience a bending load, it is often reasonable to assume that the nanowires are experiencing pure compression or tension, due to the geometry and mechanical properties of the device and substrate [58]. This results in a potential distribution along the c-direction of the nanowire, as opposed to across the diameter of the nanowire as in the case of lateral deflection.

In addition to ZnO, other materials such as ZnSnO_3_ have also been used. Wu *et al.* [58] reported a ZnSnO_3_ nanowire strain sensor demonstrating a gauge factor of 3740, the second highest reported in this review. Although less commonly studied, ZnSnO_3_ is interesting for having a calculated c-axis polarization value higher than that of ZnO [58,59,60]. The versatility of ZnSnO_3_ is further demonstrated in [57] where single microbelts are used to realize strain sensing and energy harvesting together in a self-powered device. GaN, reported to have better atmospheric and acid compatibility than ZnO, was used to fabricate a single nanobelt piezotronic strain sensor in [46]. This study also investigated the effect of c-axis *vs.* bending axis orientation using 10 μm wide GaN nanobelts [46]. Although the single ZnO nanowire configuration is common for piezotronic strain sensors, a work by Liang *et al.* demonstrates a novel approach using a tellurium microwire as the piezoelectric material [55].

#### 3.1.2. Device Architecture

The strain sensors reviewed here have a similar device architecture. A substrate, often a flexible material such as polyethylene (PET) or polystyrene (PS), serves as the base of the device [21,46,50,55,56,57,58]. A metal-semiconductor-metal structure is typically employed, with the metals serving as electrodes and forming either ohmic or Schottky contacts with the semiconducting nanostructure [21,46,50,51,53,55,56,57,58]. The Schottky contacts may be either symmetric or asymmetric, partially depending on the work functions of the chosen metals, commonly silver paste, gold, Indium-Tin-Oxide (ITO), or platinum [21,46,50,51,53,55,56,57,58]. As a final step, the nanostructures/devices are usually encapsulated in polydimethylsiloxane (PDMS) or other polymers to protect against contamination and corrosion [21,46,50,53,55,56,57,58].

One challenge to the adoption of nanowire-based strain sensors is their incorporation into devices [61]. ZnO nanostructures, for example, are commonly grown via low-temperature hydrothermal methods, or by chemical vapor deposition (CVD) [50,53,56]. The hydrothermal method is low cost, scalable, and compatible with many substrates, however, the growth rate is slow and achievement of long wires remains difficult [61]. Chemical vapor deposition results in long, high quality nanowires but is compatible with only a limited number of substrates and materials due to the high temperatures involved [62]. However, research into thin film based piezotronic devices is ongoing and shows promise [61].

#### 3.1.3. Device Characterization and Performance

The current-voltage (I-V) behavior is commonly used to characterize piezotronic devices. Typical outputs are on the order of a few to tens of microamps for biases between −3 V and +3 V [21,46,50,51,53,55,56,57,58]. The metal-semiconductor-metal structure of the devices usually has double Schottky contacts with the n-type semiconductor, however, one of the contacts may be ohmic [50]. Devices fabricated with the same electrode metal at the source and drain electrode will usually have a symmetric I-V curve while devices with different metals will have asymmetric barrier heights and display rectifying behavior [50,56]. Charge transport at the barrier is commonly explained using the thermionic emission diffusion model [21,58].

Several promising performance characteristics emerge upon examination of reported piezotronic strain sensors. These devices are often described as being stable and robust, with fast response time, high sensitivity, and high resolution [21,46,50,51,53,55,56,57,58]. Yang *et al.* estimates that a ZnO nanowire can achieve a response time of 10 ms and demonstrated frequency detection with a strain sensor by accurately characterizing AFM tip vibrations between 0.11 and 0.33 Hz [51]. Fast response times between 10 and 500 ms have been reported [21,51,53]. Gauge factor is frequently used to characterize strain sensors, and is defined as the normalized change in current divided by the strain, commonly written as

Gauge Factor= |∆II0ε|

where $I_{0}$ is the current under zero strain for a given bias, and ∆I is the change in current under that bias for strain, ε.

Gauge factors are typically provided over a range of strains for a given bias. Zhang *et al.*, achieved the highest gauge factor for piezotronic strain sensors reported in this review with a value of 4036 [53]. For comparison, a recent review of piezoresistive graphene strain sensors reports gauge factors as high as 300 [63]. The high sensitivity of piezotronic devices is a major advantage for strain sensing applications. Response time is often measured by applying periodic bending at varying frequencies under a fixed DC bias, typically between 2 and 10 V, using a mechanical stage or linear motor [21,50]. The reproducibility of the sensor is evidenced by the I-V curve returning to the unstrained state after unloading [21,46]. Although the piezotronic effect is generally not transient, current drop has been observed after several seconds during static strain measurements [58]. This is thought to be due to charge trapping by vacancies and impurities in the semiconductor [58].

The piezotronic principle readily lends itself to strain sensing applications. Strain sensors based on piezotronics are fast, sensitive, and robust. Table 1 shows a summary and comparison of piezotronic strain sensors described in this review.

**Table 1:** A comparison of strain sensing works.

Material	Morphology	M-S-M Junctions *	Gauge Factor	Response Time	Reference
ZnO	NW, array	Schottky/ohmic	1813		[50]
ZnO	NW, single	Schottky, symmetric		500 ms	[51]
GaN	NB, single	Schottky, symmetric			[46]
ZnO/Carbon Fiber	NW/fiber	Schottky, asymmetric	81		[56]
ZnSnO_3_	NB, single	Schottky, symmetric			[57]
ZnO (In doped)	NB, single	Schottky, symmetric	4036	120 ms	[53]
ZnO	NW, single	Schottky, symmetric	1250	10 ms	[21]
ZnSnO_3_	NW, single	Schottky, symmetric	3740		[58]
Te	NW, single	Schottky, symmetric Schottky/ohmic			[55]

NW, nanowire; NB, nano/micro belts/ribbons; M-S-M, metal-semiconductor-metal; *: “Symmetric/asymmetric” refers to junction material choice, actual barrier heights may differ.

### 3.2. Piezotronics for Pressure and Force Sensing

Pressure and force sensing are based on the same application of the piezotronic principle as the strain sensor; however, the quantity of interest is pressure or force instead of strain. Unlike strain sensors in which a nanowire experiences tension and compression by way of lateral bending, the nanowires in pressure sensors typically experience forces directly applied along the c-direction in the case of nanowires [64,65]. Applications of piezotronic pressure and force sensors include e-skin, handwriting recognition, and pressure sensitive electrical triggers [66,67]. Great progress has been made towards these applications, and tactile imaging has exceeded the spatial resolution of the mechanoreceptors in the human hand [66,68]. In piezotronic pressure sensors, the applied force or pressure creates a strain-induced piezopotential that controls charge transport across a metal–semiconductor junction [65]. Trends in device fabrication, materials, structure and characterization are similar to those previously mentioned for strain sensors, and both rigid and flexible substrates have been used [65,66].

A work by Wu *et al.* demonstrates pressure sensing using a piezotronic array of vertical ZnO nanowires [65]. The device was fabricated on a flexible PET substrate with ITO and gold on the top electrode and ITO/Au/Cr on the bottom electrode. A patterned ZnO seed layer allowed for the growth of vertical nanowire arrays between the electrodes, with the arrays packaged in SU-8 for robustness. SEM imaging showed the arrays to consist of 30 μm long wires in 20 μm × 20 μm pixels. The Schottky contact formed between the nanowires and gold created an addressable array of Strain Gated Vertical Piezotronic Transistors (SGVPT). The final device had a spatial density of 8464 taxels·cm^−2^, which exceeded the spatial density of 240 cm^−2^ for mechanoreceptors in the human hand [68]. Pressure sensing capability was tested by measuring the current response to increasing pressure for a single taxel. The sensor was saturated at 30 kPA, showing a pressure sensing range comparable to the human finger [69]. The calculated sensitivity of the device was 2.1 μS·kPa^−1^, which is a measure of carrier transport changes due to the applied strain and resulting potential [65]. Experimental results showed the response time was determined to be 0.15 s and that the piezotronic effect dominated the device behavior. Additionally, the handwriting applications of this sensor were demonstrated by showing the different responses of individual taxels to an imprinted letter “A”. The single crystal nanowire-based sensor is robust, with stable response after 1000 bending cycles.

**Figure 4:**
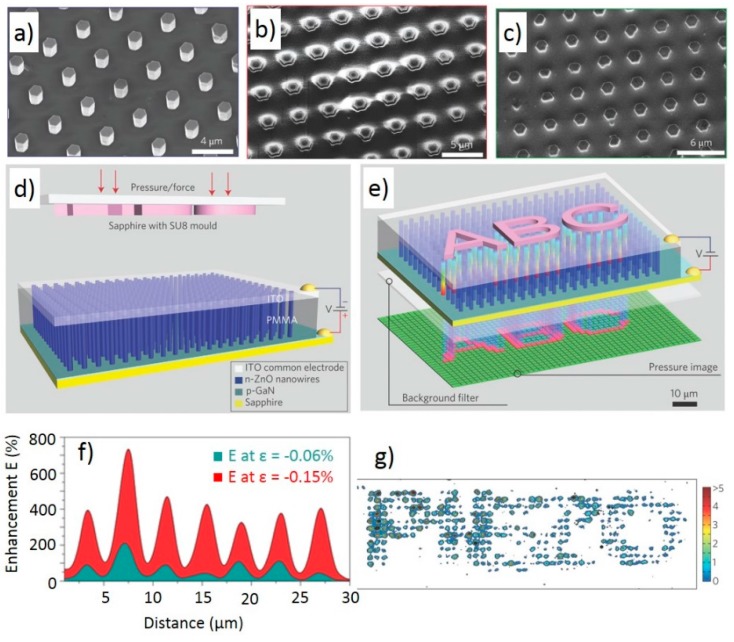
SEM images of hydrothermally grown ZnO nanowire arrays (**a**) on GaN substrate; (**b**) infiltrated with PMMA and etched to expose tips and (**c**) with ITO top electrode deposited; (**d**) Illustration of device cross section and testing procedure, and resulting image (**e**) from applied pressure; (**f**) Enhancement factor for a subset of an LED row at −0.06% and −0.15% strain. Peaks correspond to LED locations; (**g**) Spatial map of enhancement factor resulting from imprint of the “PIEZO” text stamp on the sensor. Adapted by permission from Macmillan Publishers Ltd: Nature Photonics [64] Copyright 2013.

When the piezotronic effect modulates charge transport behavior in optoelectronic devices, it is known as the piezo-phototronic effect. More commonly associated with photo detectors, the piezo-phototronic effect will be discussed later in greater detail in Section 3.4 of this review. However, the piezo-phototronic effect also finds application in pressure sensing as described in a work by Pan *et al.* [64]. In this work, ZnO nanowires were combined with GaN to create an array of pressure sensitive light emitting diodes. The device was fabricated on a p-type GaN thin film. An array of individual, vertical ZnO nanowires was grown on the device using the hydrothermal method resulting in 1.5 μm diameter wires of uniform length. Next, a bottom electrode of Ni and Au was deposited and the wires were infiltrated with PMMA. SEM images of the nanowire array before and after PMMA infiltration are seen in Figure 4a,b. The approximately 1.5 μm diameter nanowires have a pitch of 4 μm, resulting in a resolution of 2.7 μm. Lastly, a common ITO top electrode was applied by sputtering as seen in Figure 4c. Figure 4d shows a profile view of the device along with the testing setup. To validate device performance, an SU-8 mold of the text “PIEZO” was pressed onto the top of the device and the resulting output intensity of the LEDs was measured with a CCD sensor and spectrometer as shown in Figure 4e. The text pattern was applied at strains ranging from −0.06 to −0.15. The resulting LED intensity was converted to an enhancement factor defined as the intensity change due to strain divided by the undeformed intensity [64]. Figure 4f shows the enhancement factor for a portion of a single row of LEDs under different strains. The 2D spatial map of the enhancement factor is displayed Figure 4g with an average enhancement factor of 2. The use of common top and bottom electrodes allows for a response time of 90 ms.

**Figure 5:**
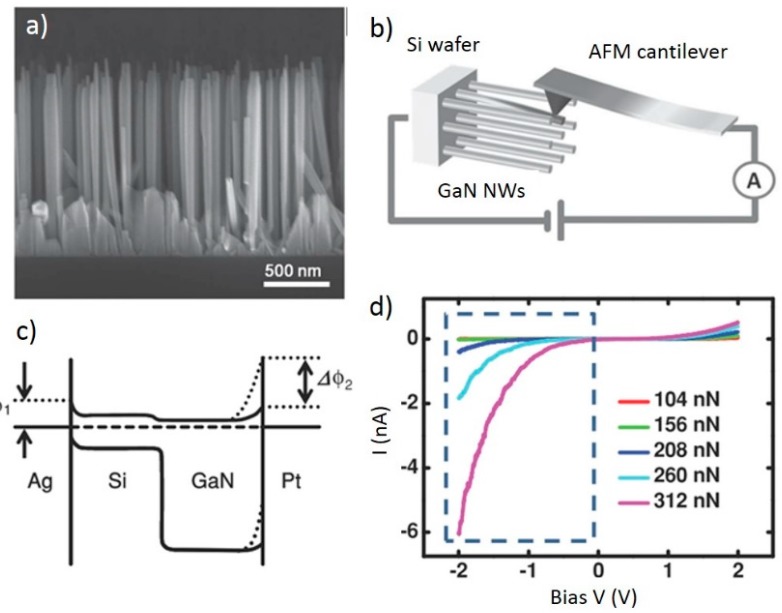
(**a**) SEM side view of vertical GaN nanowires; (**b**) Device testing schematic; (**c**) Band diagram of Ag-Si/GaN-Pt metal-semiconductor-metal architecture; (**d**) Current-voltage characteristics measured against applied force. Adapted by permission from John Wiley and Sons: Advanced Materials [66] Copyright 2012.

Beyond its use in the aforementioned pressure sensor, GaN also finds use in a transverse force sensor composed of vertical GaN nanowires [66]. Zhou *et al.*, explored the behavior of the piezotronic effect in in GaN nanowires under lateral force instead of the more commonly explored axial strain. In this work, molecular beam epitaxy was used to create an array of 1.5 μm long GaN nanowires doped with n-type Si on a silicon substrate as shown in the SEM image of Figure 5a. Using an AFM, bending force was applied to the tip of a single nanowire as seen in Figure 5b. The AFM tip was coated with platinum, creating a Schottky contact between the AFM and nanowire, while the silver paste was used as a bottom electrode creating a second Schottky contact with a lower barrier height.

Figure 5c shows the band diagram with changes in the Schottky barrier height between the AFM tip and the nanowire, which are driven by the induced piezopotential [66]. The I-V response to applied forces from 104 to 312 nN under varying bias can be observed in Figure 5d. The highly asymmetric nonlinearity of the response is consistent with the differences in the work functions of silver and platinum, and shows that the device behavior is dominated by the platinum-GaN Schottky barrier. Because of this nonlinear relationship, the sensitivity of the device is dependent on the force range. The sensitivity was found to be 0.5 pA/nN from 16 to 32 nN and 2.0 pA/nN from 64 to 80 nN. For ease of use, the sensitivity was linearized by the ln(I) to be approximately 1.24 ± 0.13 ln(A)/nN. In addition to this high sensitivity, the sensor displayed a fast response time of <5 ms, demonstrating a common and favorable property of nanomaterial based sensors.

**Figure 6:**
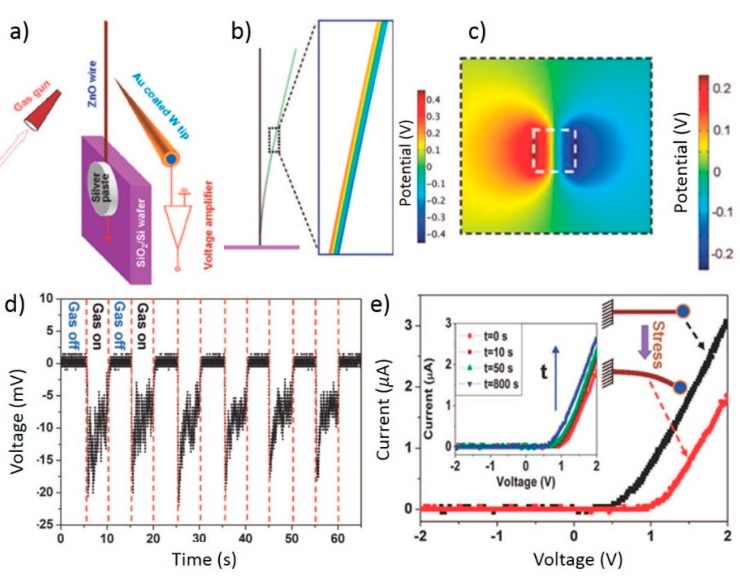
(**a**) Device schematic and experimental setup; FEM model of potential distribution along wire length (**b**) and cross-section (**c**); Dynamic response to gas pulses (**d**); (**e**) I-V behavior under zero and maximum deflection conditions. Recovery behavior of I-V plots inset for varying times after relaxation from maximum deflection. Adapted with permission from [67]. Copyright 2008 American Chemical Society.

Lastly, we review a piezotronic trigger sensor developed by Zhou *et al.*, which can be used to detect impact forces [67]. This device was based on ZnO micro or nanowires fabricated by thermal evaporation. The experimental setup consisted of a single wire approximately 1 mm long connected with silver paste to a silicon wafer coated with a layer of SiO_2_ as shown in Figure 6a. The top electrode consisted of an Au coated tungsten needle, which formed a Schottky contact with the ZnO nanowire. To excite the nanowire, argon gas was blown perpendicular to the wire, causing it to vibrate against the needle, thereby simulating an impact force. Contact between the nanowire and needle were therefore periodic, and the voltage was measured each time the needle contacted the wire. Despite lower than predicted output voltages, the system was able to resolve the periodic contacts. Because the potential distribution in a laterally bent ZnO nanowire occurred across the diameter of the wire as seen in Figure 6b,c, voltage measurements were taken on both the tension and compression sides of the wire. To measure the sensor’s dynamic response, the output voltage on the compressive side of the wire was measured under the periodic application of gas flow as shown Figure 6d. Distinct voltage changes can be seen which could be used to indicate that an impact event has occurred. Finally, the I-V characteristics of the wire were measured by applying a constant mechanical force to the wire under a bias ranging from −2 to +2 V. The red and black curves in Figure 6e show the current response at the undeformed and maximum deformation state while the inset shows the time recovery of the of the I-V curve from the deformed to undeformed state.

Like strain sensors, piezotronic force and pressure sensors also exhibit high performance with force sensing thresholds as low as 4 nN [54]. The resolution, which is force range dependent, has been reported to be as high as 0.5 pA/nN for a 16–32 nN force range [66]. Spatial resolution as high as a 2.7 μm has been achieved for piezotronic pressure sensors [64]. ZnO nanowires make for robust pressure sensors due to their single crystal nature, and no degradation was observed over 1000 bending cycles [65]. Table 2 shows a comparison of piezotronic force and pressure sensing devices.

**Table 2:** A comparison of force and pressure sensing works.

Material	Morphology	M-S-M Junctions *	Sensitivity	Spatial Resolution	Response Time	Reference
GaN	NW, single	Schottky, asymmetric	0.5–2 pA/nN		<5 ms	[66]
ZnO	NW, array	Schottky, asymmetric	2.1 μS·kPa^−1^	100 μm	150 ms	[65]
ZnO	NW, single	Schottky, asymmetric			10 ms	[67]
ZnO	NW, array	ohmic, asymmetric	12.88 GPa^−1^	2.7 μm	90 ms	[64]

NW, nanowire; M-S-M, metal-semiconductor-metal. *: “Symmetric/asymmetric” refers to junction material choice, actual barrier heights may differ.

### 3.3. Piezotronics for Chemical Sensing

Nanostructured materials have been widely explored for chemical sensing applications thanks to their high surface to volume ratio. The adsorption of chemical species on the surface of the nanostructures changes their charge carrier density, and therefore, the current flowing through the device. Thus, a large current change is desirable for a high performance. Conventional ohmic-contact-based nanowire device requires very small nanowires and low contact resistance with electrodes to obtain sufficient sensitivity [27]. This requirement presents a significant challenge for the fabrication process. Alternatively, Schottky-contact-based devices have been demonstrated to provide superior sensing performance compared to ohmic-contact-based devices regarding not only sensitivity but also resolution, signal level and response time [27,70,71]. This significant enhancement can be attributed to the tunability of the Schottky barrier height at the metal-semiconductor contact, which can be increased or decreased by the strain-induced piezoelectric potential. Similar to the sensing applications discussed in previous sections, the piezotronics effect is also utilized to shift the Schottky barrier height at the contacts to obtain the optimal current response.

#### 3.3.1. Piezotronics for Biosensors

Semiconducting nanowires used in field effect transistors (FET) are promising candidates for bio-sensing applications due to their large surface to volume ratio and ability to be functionalized to detect specific biomolecules. The piezotronic effect has been investigated to enhance the device performance of some important biosensors, such as glucose sensors [72]. The mechanisms through which the piezotronic effect enhances the sensor’s performance in the following reviewed study also apply for other biosensors [73,74,75].

**Figure 7:**
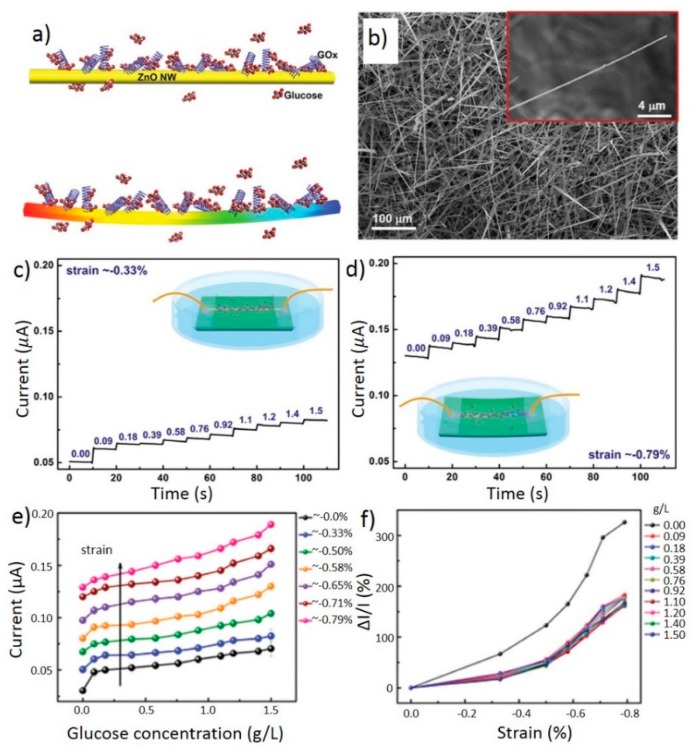
Schematic of the ZnO nanowire glucose sensor and its performance. (**a**) Unstrained and strained single ZnO nanowire functionalized with GOx; (**b**) SEM image of the as-synthesized ZnO nanowire. Inset image is a magnified view of a single wire; I-t characteristics of the single wire sensor as glucose is added over time under (**c**) 0.33% compressive strain and (**d**) 0.79% compressive strain. The glucose concentrations are in gL^−1^; Absolute (**e**) and relative (**f**) current response of the glucose sensor under different glucose concentrations, from 0 to 1.5 gL^−1^ and strains, from 0% to −0.79%. Adapted from [72] with permission from John Wiley and Sons. Copyright 2013.

The enhancement of glucose sensing FETs through the piezotronic effect was successfully demonstrated in a recent study [72]. The ZnO nanowire has been reported as a potential candidate for glucose sensing [76] and was used in this work to provide the glucose sensing function. The Schottky contacts were obtained by using silver paste to clamp the two ends of the ZnO nanowire, with c-axis pointing toward the source electrode. The two Schottky contacts were then isolated from the environment by a layer of epoxy. To functionalize the nanowire for glucose sensing, glucose oxidase (GOx) was used to decorate the surface of the ZnO nanowire. The schematic diagram of the surface treatment and an SEM image of the ZnO nanowire are shown in Figure 7a,b, respectively. The nanowires, obtained under thermal evaporation process [77], are typically hundreds of nanometers in diameter and hundreds of micrometers long. Figure 7c,d shows the current response over time as glucose is added under 0.33% and 0.79% compressive strains, respectively. The current increases quickly after the addition of glucose. As the compressive strain becomes higher, this increase is enlarged, and the current level is also shifted to higher values. These improvements in current response indicate that the resolution and the signal to noise ratio of the sensor are enhanced by simply applying higher compressive strain to the nanowire. More systematic measurements of the dependence of the current on the applied strain and glucose concentration are shown in Figure 7e,f. The increasing slopes and signal levels of the curves under various strains verify the improvement of the resolution and signal to noise ratio of the sensor. Figure 7f also shows the relative change of the current increases up to 150% under 0.79% compressive strain, which demonstrates a significant improvement in sensitivity.

The observed increase of current by adding glucose is the result of higher charge carrier density produced by GOx-catalyzed glucose reaction with water and oxygen on the ZnO surface. In this reaction, H_2_O_2_ is produced and injects electrons onto the n-type ZnO nanowire, increasing its conductivity [72]. This response can be further enhanced by strain through the piezotronic effect at the reversed-bias Schottky contact, where the Schottky barrier is dominant in the charge transport process throughout the device. With the c-axis pointing toward the source and the nanowire being under compression, non-mobile ionic positive charges accumulate at the drain and tune the Schottky barrier height at the interface between the n-type ZnO nanowire and silver. Since the current response is exponentially dependent on the barrier height, it can be significantly tuned by the applied strain to the high sensitivity range for glucose sensing as demonstrated by the experimental results.

#### 3.3.2. Piezotronics for Gas Sensors

Another important application of FET-based sensors is oxygen sensing, which can find many markets such as life protection or automobile engine control. As in other chemical sensing applications, nanowire-based devices are promising candidates due to their high sensitivity and low power consumption [23,78]. In a recent study [24], ZnO nanowire was selected to demonstrate the performance enhancement via piezotronic effect thanks to its dual semiconducting and piezoelectric properties. The mechanism through which the piezotronic effect enhances the sensor’s performance in the following reviewed study also apply for other gas sensors such as H_2_ or NO_2_ sensors [79].

**Figure 8:**
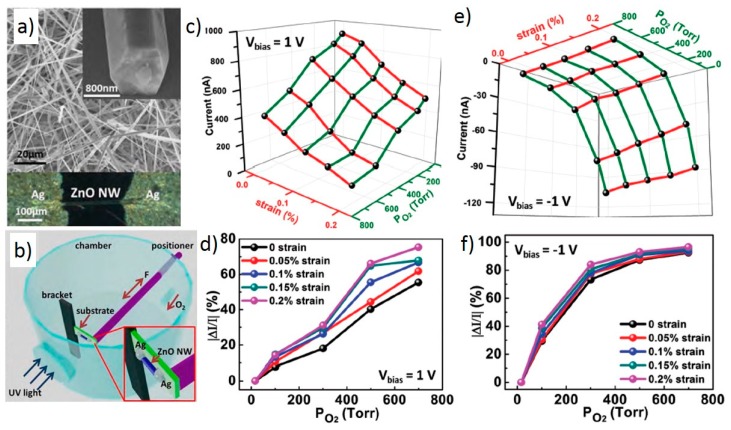
ZnO nanowire oxygen sensor and its performance. (**a**) SEM image of the as-synthesized ZnO nanowires. Inset image is a magnified view of a single wire. The bottom half is optical microscopy image of a typical ZnO nanowire oxygen sensor device; (**b**) Schematic of the measurement setup for studying the piezotronic effect in a ZnO nanowire oxygen sensor; (**c**) 3D graph depicting the current response under different strains and oxygen pressures at a bias of 1 V; (**d**) Magnitude of relative current change with oxygen pressure under different tensile strains from 0% to 0.2% at a bias of 1 V; (**e**,**f**) are corresponding results of (**c**,**d**) at a bias of −1 V. Adapted from [24] with permission from John Wiley and Sons. Copy right 2013.

ZnO used in this work was synthesized using the thermal evaporation method[24,77] . A single nanowire with 800 nm diameter and 200 μm length was transferred onto a flexible PET substrate and clamped by silver paste at its two ends to obtain Schottky contacts. The as-synthesized nanowires and the device are shown in Figure 8a. The characterization of oxygen sensing performance was carried out in a vacuum chamber, which is shown in Figure 8b where the oxygen pressure can be controlled. A positioner inside the chamber was connected externally to apply strain on the PET substrate. The nanowire was treated with UV light through a quartz window on the chamber’s wall to remove residual oxygen on the device. Figure 8c–f shows the performance of the oxygen sensor under different strains and bias voltages. The distinct difference of the current response under +1 V and −1 V biases, shown in Figure 8c,e, respectively, indicates that the device response was governed by the Schottky barrier. The performance enhancement of the piezotronic effect was studied by extracting relative current change from Figure 8c,e, which is shown in Figure 8d,f. The results clearly demonstrated that the relative current change was significantly improved, as much as from 40% to 70% under 0.2% tensile strain and 1 V bias. Under −1 V bias, the tensile strains were still beneficial although to a lesser degree due to the differences of Schottky barrier heights between the two ends of the nanowire.

The drop of the current can be explained by the adsorption of oxygen on the ZnO nanowire surface, which formed an electron depletion layer and reduced the charge carrier density in the nanowire. The adsorption of oxygen at the interface also increased the Schottky barrier height and contributed to the current drop. This current drop was further improved by piezoelectric charges created when the nanowire was under tensile strain. At the reversed-bias Schottky contact, the negative charges increased the Schottky barrier height, causing the current to drop much further due to the exponential relationship between current and barrier height. Therefore, the utilization of Schottky contact and piezotronic effect to tune the Schottky barrier height played the key role in improving the sensitivity of the sensor.

### 3.4. Piezotronics for Photodetectors

The piezotronic effect can also be combined with photon excitation processes to tune their optoelectronic performance. This three-way coupling effect among piezoelectric, semiconductor and photon excitation is called piezo-phototronics [80]. In piezo-phototronics-enhanced devices, piezoelectric polarization charges formed at the Schottky contact or p-n junction modify the photon-induced electron-holes pair generation, separation and recombination. Therefore, a superior photoresponse can be obtained compared to conventional optoelectronic devices which rely only on two-way coupling between photon excitation and semiconductor.

Silicon-based photodetectors are important for various applications due to their high compatibility with the integrated circuit technology. Novel strategies are needed for conventional optoelectronic devices to improve the photosensitivity. Wurtzite structures such as ZnO are piezoelectric semiconductors and, therefore, potential candidates for piezo-phototronic devices. A recent study [81] has demonstrated the piezo-phototronics effect as an effective approach to optimize silicon-ZnO-based p-n junction photodetector. Similar principles are also applied for photodetectors using other material systems based on various piezoelectric semiconductors, such as ZnO [82,83,84,85,86], CdS [87], Mg_x_Zn_1-x_O [88], and ZnTe [89].

**Figure 9:**
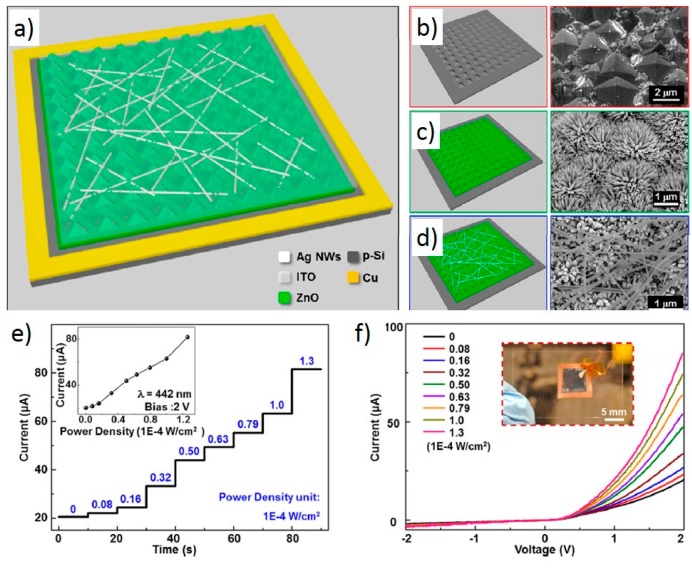
Device fabrication and its performance without strain. (**a**) Schematic structure of a p-Si/n-ZnO nanowire hybridized photodetector; (**b**–**d**) Schematic structure (left panel) and SEM images (right panel) of (**b**) etched Si wafer and n-ZnO heterojunction (**c**) before and (**d**) after spin coating silver nanowire; (**e**) I-t response of the device under different illumination power densities at 2 V bias voltage. The inset shows the current change with power densities; (**f**) I-V characteristics of the device under different illumination power densities when a triangle wave is applied. The inset shows the real device. Adapted from [81] with permission from American Chemical Society. Copyright 2014.

The photodetector was fabricated by first patterning a p-type silicon wafer with micro-pyramids to increase surface area for light absorption. N-type ZnO nanowires were grown along the c-axis on the patterned ZnO seed layer on the silicon substrate to create the p-n heterojunction. ZnO nanowires serve as the piezotronic structure as well as the antireflection layer. Silver nanowires and a layer of ITO were then deposited on the ZnO nanowire structure to serve as the top electrode. The schematic of the device and the nanostructures are shown in Figure 9a–d.

**Figure 10:**
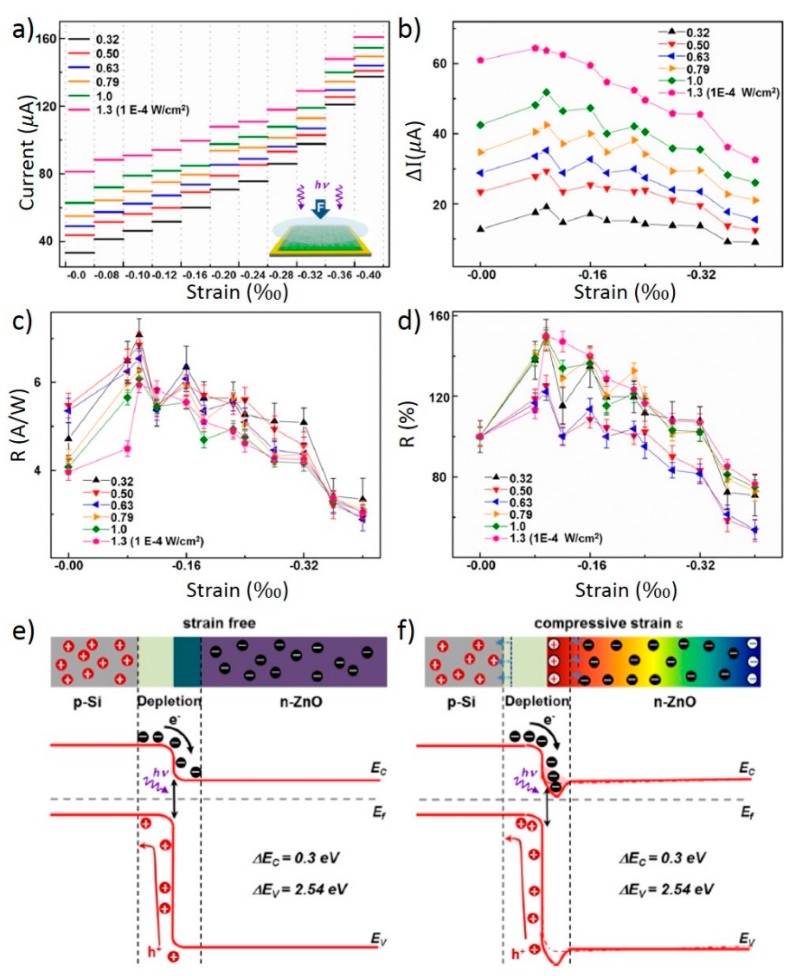
Piezo-phototronic effect. (**a**) Output current of the photodetector under different strain and illumination conditions at 2 V bias. The inset is a schematic of experimental setup; (**b**) Photocurrent; (**c**) photoresponsivity; and (**d**) relative change of photoresponsivity of the device under different strain and illumination conditions at 2 V bias; (**e**,**f**) Schematic band diagrams of a p-Si/n-ZnO heterojunction (**e**) without and (**f**) with compressive strain applied to illustrate the working mechanism of piezo-phototronic effect of the optimized p-n junction photodetector. Adapted from [81] with permission from American Chemical Society. Copyright 2014.

The materials on one side of the interface were transparent to the photons. The photoexcitation happened preferably at the interface and it can be tuned by the piezo-potential. Considering the bandgaps of the n-ZnO and p-silicon, photons will pass through the n-ZnO layer and are mostly absorbed at the interface. The general photoresponses of the device are shown in Figure 9e,f. Clearly, higher power density of the 442 nm-wavelength photons caused a higher current due to more electron-holes pairs created at the interface. The compressive strain was applied by pressing the surface of the device by a piece of sapphire. The effect of the applied compressive strain is shown in Figure 10a–d. Figure 10a shows that compressive strain increases the current level for all illumination power density. The relative change of current, photoresponsivity (R) and relative change of photoresponsivity in Figure 10b–d all follow a similar trend, which exhibits a maxima at −0.10% strain. The maximum value of R at 7.1 A/W is reached at 3.2 × 10^−5^ W/cm^2^, corresponding to a relative enhancement of 177%. The applied strain was also shown in this study to have no significant effect on its inherently fast response time.

The observed maximum in R can be explained by the competing effects of the widening of the depletion region within the p-silicon and the electron-trapping by the piezo-charge within the n-ZnO, which is illustrated through the band diagrams in Figure 10e,f. Comparing Figure 10e,f, a wider depletion region along with the dips in energy level at the interface, which represents the charge-trapping effect, are the simultaneous results of the piezo-charges in n-ZnO. Because the c-axis of ZnO nanowire pointed outward from the silicon surface, the compressive strain would cause positive charges to accumulate at the p-Si/n-ZnO interface, within the ZnO side. These positive charges cause the widening effect of the depletion region in the p-silicon side, which provide more volume for the electron-hole pairs to be formed by incident photons. However, increasing positive piezo-charges as the compressive strain increases also trap electrons at the interface and reduces the current. These two effects yield the maxima observed in Figure 10b–d.

### 3.5. Piezotronics for Temperature and Humidity Sensing

Many mechanical-type temperature sensors are based on the thermal expansion of matter, and one example is the alcohol thermometer. Because the piezotronic effect is a sensitive mechanism to measure strain, including thermal strain, it may be integrated with mechanical temperature sensors to simplify the sensor readout and improve their sensitivity.

**Figure 11:**
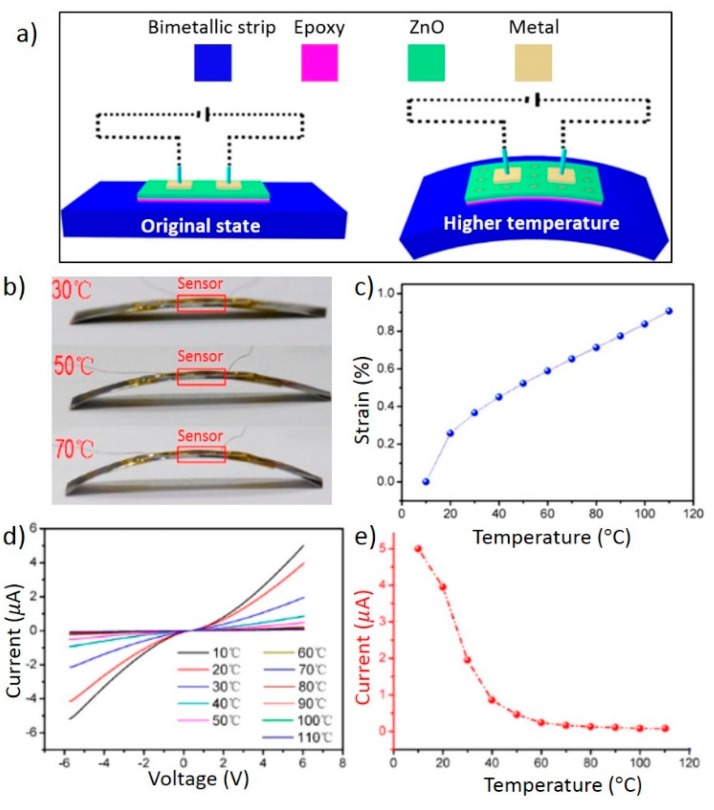
Piezotronic temperature sensor. (**a**) Structure of the temperature sensor and its working principle; (**b**) Photographs showing bending of the temperature sensor at different temperatures. The bimetallic strip was 5.5 cm in length and 2 cm in width; (**c**) Relationship between ambient temperature and strain on the surface of the bimetallic strip; (**d**) Current-voltage curves of the temperature sensor at different temperatures. The current amplitude decreased as the temperature increased; (**e**) Current of the sensor at different temperatures, with the bias fixed at 6 V. Reprinted with permission from [90] (Copyright 2014, American Chemical Society).

Xue *et al.* combined a piezotronic device with a bimetallic strip for temperature sensing [90]. The working principle is illustrated in Figure 11a. A ZnO textured film was bonded to a bimetallic strip by epoxy, and two silver electrodes were deposited on the ZnO film to form Schottky contacts. As the temperature increased, the bimetal bent and caused in-plane strain in the ZnO film. Negative piezoelectric charges were induced on the surface of ZnO and raised the two Schottky barriers, thus reducing the current flow.

For device fabrication, a ZnO textured film was first synthesized on a flexible substrate via a 90 °C hydrothermal reaction and then transferred onto a bimetallic strip [90]. Direct synthesis of a ZnO film on a bimetal was not feasible, since the bimetal readily deformed in the heated growth solution. Figure 11b shows the final device and subsequent bending at different temperatures. Figure 11c shows the relationship between temperature and strain on the surface of the bimetal, which is approximately linear in the range between 20 and 110 °C.

In Figure 11d, current-voltage curves of the piezotronic temperature sensor were measured at different temperatures, from 10 to 110 °C. Each curve is nonlinear but roughly symmetric. That is because the two Schottky barriers were almost the same after device fabrication, and strain-induced piezoelectric charge density was also uniform on the film surface. As the temperature increased, the current decreased. Figure 11e shows the current at a fixed bias of 6 V. Therefore, the current value provided direct measure of the temperature. Sensitivity of the device depended on both temperature and driving voltage. In the most sensitive temperature range with 6 V bias, one degree Celsius increase in the temperature led to a 3% decrease in the current [90].

**Figure 12:**
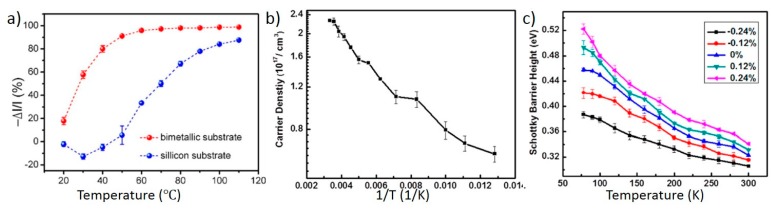
Direct effect of temperature on material properties of piezotronic devices. (**a**) Normalized current change at different temperatures, from two devices fabricated by Xue *et al*. The red curve is from an original piezotronic temperature sensor with a bimetal substrate. The blue curve is from a contrast device with a silicon substrate. Reprinted with permission from [90] (Copyright 2014, American Chemical Society); (**b**) Relationship between carrier density and inverse temperature in a piezotronic device fabricated by Hu *et al*.; (**c**) Change of the effective Schottky barrier height at one contact as the temperature decreased, at different strain levels. Reprinted with permission from [91] (Copyright 2013, American Chemical Society).

It should be noted that temperature not only induced the piezotronic effect through the deformation of the bimetal strip, but also changed the material properties of ZnO, such as carrier density and mobility. To understand the influence from the material property change, Xue *et al.* fabricated a contrast device in which the metallic strip was replaced by a silicon substrate while all the other components were the same as the original device [90]. Since silicon has a low thermal expansion coefficient, the piezotronic effect was considered insignificant in the contrast device. Figure 12a compares an original device with a contrast device. The vertical axis represents the normalized current change at different temperatures; it is defined as $-∆I/I=\left(I_{10{}^{\circ}C}-I_{temperature}\right)/I_{10{}^{\circ}C}$, where $I_{10{}^{\circ}C}$ is the current at 10 °C. With a silicon substrate, as the temperature increased, current first increased slightly and then decreased, which can be attributed to the change of material properties of ZnO. With a bimetallic substrate, the current decreased more rapidly as the temperature increased, and the difference between these two curves represents the contribution from the piezotronic effect.

Hu *et al.* also studied the temperature dependence of material properties in a piezotronic device [91]. The device was a single crystal ZnO nanowire with two silver electrodes as Schottky contacts. I-V curves of the nanowire at different temperatures and strains were measured. By fitting the I-V curves to a metal-semiconductor-metal transport model, semiconductor parameters such as carrier concentration and effective Schottky barrier height can be obtained [92]. As shown in Figure 12b, when the temperature decreased from 300 to 77 K, the carrier density in a nanowire decreased from 2.2 × 10^17^ to 6 × 10^16^ cm^−3^ due to donor freeze-out. Figure 12c shows the Schottky barrier height at different temperatures; regardless of the strain applied to the nanowire, the barrier height always increased as the temperature decreased from 300 to 77 K. This can be explained by the thermal field emission model of the Schottky contact. At a lower temperature, carriers have less kinetic energy to overcome the Schottky barrier, and equivalently the barrier seems thicker/higher to the carriers; in addition, a lower temperature may also change the occupation of surface states at the interface. The implication of this research is that, without strain, temperature can still affect the current transport of a piezotronic device by changing the carrier density and effective Schottky barrier height, and those effects must be taken into consideration when interpreting the data from a piezotronic temperature sensor.

The working mechanism of the piezotronic temperature sensor applied also to other sensing applications as long as the input physical stimulus can induce mechanical deformation. For example, moisture can cause swelling in many polymers; an old type of humidity sensor is based on the length change of an animal hair as it absorbs moisture. Such humidity-caused strain may be measured by a piezotronic device to indicate the humidity level. It is also worth noting that unlike temperature, humidity does not directly affect the Schottky barrier height of the piezotronic device [93], and, therefore, the data interpretation may be simpler.

## 4. Conclusions/Outlook

In this article, we reviewed the sensing applications in the fast developing field of piezotronics. The piezotronic effect provides a novel approach for modulating junction properties through mechanical stimuli. Various sensors have been demonstrated with much enhanced performance thanks to the exponential dependence of the carrier transport on the barrier height at the interface. Development of piezoelectric semiconducting nanomaterials has a bright future for creating multifunctional sensors. However, practical application of this new technology asks for more research efforts to address challenges that remain. First of all, cost needs to be reduced and the yields and controllability need to be improved for nanomaterial growth and device fabrication. Second, the piezoelectric polarization change can be induced from pressure, thermal strain, absorbed species, *etc*. The interactions are intertwined, so the selectivity and stability of the sensor needs to be investigated. Third, the piezoresistive effect also causes conductivity change in response to the mechanical strain, and both the piezoresistive and piezotronic effects need to be carefully studied in the design and characterization of sensors [45]. In addition, the development of new piezotronic nanomaterials and novel sensors are expected in the future. Finally, the integration of multifunctional sensors with other electronics and nanogenerators will be critical for the realization of self-powered nanosystems.
